# The Dentist’s Hygiene

**Published:** 1892-06

**Authors:** E. De Trey

**Affiliations:** Basel, Switzerland


### THE DENTIST'S HYGIENE.[1]

BY E. DE TREY, D.D.S., BASEL, SWITZERLAND.

Gentlemen,—I do not remember meeting with any treatise bearing directly on the subject of the *dentist's own hygiene*, though there are several bearing upon dental hygiene. This is certainly a very important question. The loss of health too often incurred by the long-continued practice of dentistry is a sad proof of the necessity

---

[1] Read before the American Dental Society of Europe, at Heidelberg, August, 1891.

of this article. The dentist must keep his health for his own sake and that of his clients. Operating from day to day produces a heavy strain on the vital forces, and induces great nervous tension in the operator.

What a pity to see a man practising his art under unfavorable conditions!

The digestive and respiratory organs suffer from the abnormal position of the body; the nervous system continually strained, like the cord of a bent bow, ceases at last to perform its functions, and finally the operator tires of his vocation, while his work loses its former excellence.

It is only by wisely regulating the details of his life that the practitioner can attain clear and intelligent conceptions, rapid and easy execution, as well as find pleasure in the performance of his duties. Long is the list of men engaged in intellectual or manual pursuits who, by neglecting the laws of hygiene, have seen their strength decline, and, cut down prematurely, they have not fulfilled the hopes entertained of them, nor furnished that career which they themselves had traced out.

This abstract is offered only to the serious members of our profession, and not to those whose ignorance and incapacity lower and degrade it to the level of charlatanism. I shall attempt to perform my task as well as I can, and as far as an experience of twenty-three years will enable me.

To practise dentistry it is necessary to have good health and a strong constitution, plenty of muscular force, and an active, nervous temperament, because a phlegmatic man can never make a good operator. By means of hygiene, it is not only possible to keep one's self up to the mark, but also to make up for one's deficiencies, in the one case by a careful diet and healthy habits, and in the other by a painstaking and methodical manner of operating.

The questions of situation, light, ventilation, and heating are all important ones to the dentist's health. Considering that he has to pass a great part of his time in-doors, a gay and sunny apartment will have a beneficial effect on his character. The operating-room must be so separated from the waiting-room that the cries, groans, or conversation of the patient cannot be overheard. The proximity of the two would distract the operator, and increase his nervous tension, which is bad for all concerned.

The laboratory must also be at some distance from the operating-room, without being out of reach. An equable temperature must reign in the different rooms, because the operator is likely to cool down quickly after heavy work. The temperature ought to be about 20° C. He who works in-doors requires a higher temperature than he who works out of doors; the latter takes more exercise, and the natural heat thus developed is increased by more abundant feeding. On the other hand, overheated air draws the blood to the brain, where it is always attracted by intellectual effort. The temperature must be constant, else the operator, absorbed in his work, may not notice its variations. The operating-chair should not be too near the fire. Moreover, as much as possible, let the windows remain open, as fresh air is highly beneficial to health. An open chimney with a wood-fire is best in the operating-room, because this system constantly renews the air; a stove will do for the rest of the apartment. A northern light is best for the sight, on account of its steadiness; and western light is less blinding than an eastern one. The most valuable operating is done in the morning hours, when the body has been refreshed with sleep. A southern light is bad on account of its unsteadiness and the heat from the sun's rays. If the light is reverberated by a sheet of water below the window it becomes very vacillating. A blind working upward will greatly lessen this evil, which is very prejudicial to the sight.

The dentist practising in a dark street, or in a misty country such as England, will find a white blind working from below upward very useful.

In choosing an operating-room, attention must be given to the size, and especially to the height of the windows, as well as to the size of the room, which ought to be spacious. It is easy to understand that plenty of air and light conduce to better work than a restricted amount of these.

A continual subject of discussion is the point whether the dentist's residence should be at or away from the spot of his labors. The author has tried both plans. Keeping up separate apartments is expensive, but a counterbalancing benefit is the necessity of walking to and fro, and thus getting needful exercise and a change of ideas. The practitioner who simply leaves his operating-room to pass into an adjoining one for his meals, is liable to lose his appetite, get enervated and cross, and his food will not profit him as it should. If he be not forced to take out-door exercise, he will often become neglectful of it, and the less he takes, the less he will wish to take. Patients knowing that a man resides where he practises will often insist on seeing him just for a moment at undue times and in spite of orders against their admission. The combination of residence and office is useful for saving time, for easier work and study during the winter evenings. On the other hand, the dentist is thus more easily distracted by the ties of family life. All things considered, the author is of opinion that the advantages of enforced exercise ought to overrule all other consideration. Great cleanliness must reign all over the apartment. The laboratory should have a chimney to carry off all acid and vulcanizing smells and vapors, which rust and corrode metal substances lying around. For the same reason, the apartment must be well ventilated morning and evening. In short, he who takes pride and pleasure in his calling will have an inviting, comfortable, and even stylish interior, so as to please clients, and even draw compliments from them.

The operator must be very careful about his personal appearance; he must have a special working-coat or jacket, to avoid carrying with him tobacco and other odors. A white, easy-fitting garment is desirable. In winter a warm Jæger coat is practical, though the best thing is white cloth or smooth silk, which both look and feel clean and neat. Black coats easily become greasy by contact with the patient's hair, or soiled by the cosmetics which ladies sometimes use.

It is difficult to lay too much stress on these niceties, which are much appreciated by the better class of clients, and the observance of which tends to build a good practice. Footwear must be soft and warm in winter, light and fresh in summer; special pairs being kept for exclusive use in the dental office. A low shoe of good calf, flannel- or fur-lined, is best in cold weather. Prolonged standing is fatiguing, therefore the dentist should have his feet easy and comfortable. A warm foot-bath after the day's work is over is often beneficial, for it promotes the circulation and relaxes the nerves. Intellectual effort draws the blood to the head, and the feet get cold in consequence, a thing to be avoided, as warm feet and a cool head insure good digestion.

Those who perspire freely from the feet should change socks once or twice a day. The writer would here remark that he has known two cases in which, perspiration having been artificially stopped, a dangerous illness was the result, followed by death. Elastic boots are apt to stop circulation, but low shoes allow the escape of heat and moisture, and are not conducive to cold feet.

The old saying of warm feet, cool head, and loose waist is to be remembered and practised. The neck also should be free, without a high or narrow collar. The pressure of a hard collar on the veins of the neck during the various flections of the head brings on congestion of the brain; and this is very bad in the case of thick-necked men with apoplectic tendencies. A fine white flannel, with loose silk tie, would be best, if etiquette allowed it.

A dentist must have perfectly clean and well-kept hands, with nails not too long. Patients have complained to us of having been scratched by too long nails, and one lovely client made us once pare our nails on the spot. Yet it is necessary to keep those of the thumb and forefinger long enough to pick up quickly the various little implements in daily use. Another point is, that an operator should not in general use too-highly-scented soap; in fact, exceptional cases apart, scent of any kind is out of place. The hands should be washed in the patient's presence, and every time the dentist is called out of his room.

When hands and arms begin to feel nervous twitchings, the best thing is to plunge them up to the elbow in lukewarm water. Should there be tension, congestion, or weariness of the brain, a cold bath will act very well. The handling of steel instruments produces horny fingers, which lose their tactile sensibility. To regain this, rub the fingers on pumice-stone, without taking off too much skin, as a certain amount of hardness is necessary.

Above all, the dentist's own mouth must be in a fit and proper condition; a bad breath is sufficient to drive away patients. There are means of rapidly sweetening the breath, but they are unwholesome to the stomach. Here are a few useful ones: Chew a slice of lemon with the rind on; this will freshen the breath for a fairly long time. The smell of garlic disappears by chewing parsley and swallowing its juice. A few drops of "hypochloride of soda" (liqueur de Labarraque) in half a tumbler of water constitute an effective and powerful deodorizer. The too frequent use of aromatic extracts, such as catechu, cloves, peppermint, etc., may injure the stomach. Catechu, for example, is an astringent which contracts the walls of the stomach, often producing cramps the origin of which is unsuspected. Cloves cauterize the mucous membrane, and deprive it of its smoothness. Generally speaking, all these substances injure the sense of taste. Chewed coffee-beans adhere to the lining of the stomach in noxious deposits. Coffee is an excellent antidote against alkaloids; a spoonful will mask their smell and annul their effects, used internally or as a gargle. Coffee and milk for breakfast is nutritious, laxative and disinfectant.

The posture of the body whilst operating, as will be shown further on, has considerable influence on the health. It is rare to find a dentist who knows how to place his patient in the chair so as to facilitate his own work and avoid mutual torture. While possessing all necessary means for working with comfort to themselves, why will not dentists take the trouble to put their patients in a position easy for both parties, instead of going through a sort of gymnastics hurtful to the eyes, lungs, and stomach? The right eye of a dentist who does much gold-filling, after fixing the same brilliant spot for hours, gets tired and becomes weak long before the left one. Deviation of the visual rays is an affection which almost always affects the right eye, and is caused by the ocular globe being twisted away from its normal position, when some muscles are overstrained and others relaxed; this habit in time dims and weakens the vision. The best and simplest way to avoid this trouble is to so place the patient in the chair as to bring all available light on the point of operation, and to look at it in a straight line, and not from a slanting direction. Also, one must learn to operate on both sides of the patient, and from behind as well as in front. By means of these different positions the visual rays enter squarely into the eye in its normal and unstrained position, and not sideways, and both eyes work equally. These methods of operating are soon learned, and their advantage quickly perceived. Operators will do well to think of the means of preserving their eyesight while still young, before becoming slaves to spectacles. Their usage is a source of annoyance to the practitioner, because the patient's breath clouds them, causing loss of time in removing and wiping. A good magnifying-glass should always be at hand, to avoid straining the sight looking at small objects, such as burr-heads, for instance. Blue glasses are good for softening glare and change of light. The means of bettering the sight when it begins to fail are numerous, but the simplest and best are rest and refreshing sleep, such as hygiene and exercise produce; reading at night, indulgence in alcohol and tobacco must be given up. Electric, petroleum, and all lamps with too powerful a glare are to be avoided; the old moderator lamps are the best. Few organs of the body are so sensitive to the diseases of the stomach as the eyes. Dimmed and vacillating vision, pain behind the eyeballs, watering eyes are almost always symptoms of a disordered stomach. Rose- or chamomile-water makes an excellent eyewash; an infusion of rosemary in good cognac brandy is good for frictions around the orbit, the brow, temples, and neck. As a rule, let us not turn night into day, but, returning to the ways of our forefathers, go to bed and rise early. They knew not the use of spectacles before old age, nor did they need the various exciting modern condiments.

In the course of numerous excursions in the Alps, the author has made some curious observations on the changes going on in the country at large. With the advent of coffee, sugar, spices, and alcohol in the most secluded valleys, the hitherto vigorous and healthy inhabitants have begun to lose their fine teeth and flowing hair. All sorts of infirmities, unknown before, have appeared among these hardy mountaineers. The author is quite convinced that this is due to alterations of the blood, brought about by exciting foods. Let us leave hot things to hot lands, and cling to what nature grows in our temperate zones. The first effect of these stimulants seems to be renewed life, vigor, and keenness, but their continued use brings in time prostration and even loss of health. Such is also the case with plants transferred from their own natural soil into a richer one: they thrive, increase in size and beauty, then droop and die away. Nervous diseases and folly count more victims nowadays than formerly in town or country, thanks to bodily and mental excitement and fast living. Let us work quietly day by day, not peering too far ahead into the future. Thus, money will be more slowly acquired, but the faculty of enjoying it in old age will be preserved. On hygiene depends the surety and suppleness of the hand, the clear vision of the eye, and on these does the dentist himself rely. Delicacy of touch is a gift, which can be developed, but not acquired; few possess it, and those who do should not imperil it by excess of any kind. It can be cultivated from infancy and maintained through life by manual exercises. This attribute can be unconsciously lost by excesses. The abuse of wines, spirits, and tobacco leads to that of narcotics, which doctors and dentists have within their reach. We cannot enough warn operators against being tempted to their use to soothe wearied nerves, for no human words can tell the sufferings to be encountered before the habit is broken. Unfortunately, numbers of medical and dental men use these drugs themselves, though they forbid them to their patients. Here are the words of a specialist in nervous and mental diseases, Dr. Foree, of Zurich: "How great a number of men are lost to science by the use of alcohol! Even what is called a moderate amount is sufficient in time to alter the tissues and weaken the mental faculties." Total abstinence is more profitable than simple temperance. If the thoughts and actions are not so quick, they are all the more reliable; fatigue and ailments have less hold on the total abstainer.

The strength and suppleness of the hand depend on that of the body. All exercises of sport and gymnastics, abundant in youth, more moderate in manhood, are a boon to the body. Combine these with hydrotherapeutics, and you have the secret of good health and good humor, wherewith to perform daily work easily and pleasantly.

Half an hour should be given every morning to bedroom gymnastics on the Swedish system, adding cold or tepid baths according to individual natures, or simply sponging the upper part of the body. These exercises must be done slowly, and in a measured way, not with rapid and unsystematic movements. To undergo a hydrotherapeutic cure in a first-class establishment is for a run-down and overworked dentist the best means of recovering health and condition. Unfortunately, these good establishments are by no means common, and we only know one, that of "Schœnbrunn," in the Canton of Zug, where the basis of the treatment consists in the avoidance of all stimulants, as alcohol, tea, coffee, pepper, spices, etc.

The dentist must not only exercise his arms and legs, but his wrists and fingers separately. It may happen that he who has not been in the habit of practising gymnastics from infancy may feel tired at first; the best plan is, then, to exercise for four or five minutes in the beginning, gradually increasing the length of the periods up to one-quarter of an hour. Frictions with rosemary and spirits strengthen and keep up the suppleness of the arms; not to mention that this old remedy is good for rheumatism. Finally, the best way to counteract the effects of a too-sedentary life is to take all possible exercise between working-hours.

What one should eat is a subject of some importance, and it is to be supposed that every one knows best what suits him, yet such is not the case. A fair meal after morning exercise is necessary to do four hours' work, and a light mid-day lunch is better than a full dinner, which makes one feel heavy and lazy. Owing to bodily fatigue and nervous tension, the dentist is at times liable to a sensation of weakness and loss of energy; this is due to a momentary lack of nourishing substances. Here Hygiene steps in and says, Stop and replenish the motor; or, in other words, take some light food,—whereupon the sinking sensation will disappear. A drop of soup about ten o'clock, a cup of tea and a biscuit about four, is all that is needed to revive the flagging energy of body and mind. The general appetite for regular meals will in no ways be injured by this habit, which is a national custom in several countries, chiefly in the North. This plan helps to avoid excess at any one time. For those men who are obliged to subject their bodies to postures which prevent the free play of the internal organs, frequent and spare meals will help to facilitate easy digestion. Plenty of milk is an excellent means of calming nervous irritation.

Sleep is the chief factor in general and dental well-being. Loss of sleep means loss of strength ; no sleep, no work. By a hygienic mode of life the act of sleeping becomes a source of pleasure, a delicious close to the day's work.

To obtain deep, calm sleep, without dreams or nightmare, total abstinence, or, at least, a very limited use of alcoholic stimulants, must be the rule. Less noxious but still powerful stimulants, such as tea and coffee, must be avoided as the night-hours draw on.

But contentment of mind comes not only from a hygienic life, but from a feeling of confidence in the future, and where this source of trustfulness is to be found, you all know. If I here enter the domain of moral and intellectual hygiene, it is because I have convinced myself of its power on the nervous functions. The sentiment of religious duties performed sincerely is an elixir without a rival, and an efficient soporific. There is the secret of a contented and happy life. As meals should take place with regularity, so should sleeping-hours be regular.

We would utter a warning-cry as to the necessity of timely relaxation from continuous toil,—to all those who are carried away by duty, love of work or money, and who labor on for years without taking the necessary holiday. As soon as the dentist takes stimulants to keep up his flagging strength, he is overstepping the limit and working too hard. Each practitioner has, according to his strength, a limited number of years before him. Therefore, let every one starting in the profession understand before it is too late that he must make his position more or less rapidly; for few vocations entail so much fatigue, wear, and tear as genuine and artistic dentistry.

